# *Urtica dioica* and *Dodonaea viscosa* leaf extracts as eco-friendly bioagents against *Alternaria alternata* isolate TAA-05 from tomato plant

**DOI:** 10.1038/s41598-022-20708-4

**Published:** 2022-10-01

**Authors:** Said I. Behiry, Bassant Philip, Mohamed Z. M. Salem, Mostafa A. Amer, Ibrahim A. El-Samra, Ahmed Abdelkhalek, Ahmed Heflish

**Affiliations:** 1 Agricultural Botany Department, Faculty of Agriculture (Saba Basha), Alexandria University, Alexandria, 21531 Egypt; 2 Forestry and Wood Technology Department, Faculty of Agriculture (El-Shatby), Alexandria University, Alexandria, 21545 Egypt; 3 Plant Protection and Biomolecular Diagnosis Department, ALCRI, City of Scientific Research and Technological Applications, New Borg El Arab City, 21934 Egypt

**Keywords:** Biological techniques, Microbiology, Plant sciences

## Abstract

One of the tomato’s acutely devastating diseases is *Alternaria* leaf spot, lowering worldwide tomato production. In this study, one fungal isolate was isolated from tomatoes and was assigned to *Alternaria alternata* TAA-05 upon morphological and molecular analysis of the ITS region and 18SrRNA, *endoPG*, *Alt* a1, and *gapdh* genes. Also, *Urtica dioica* and *Dodonaea viscosa* methanol leaf extracts (MLEs) were utilized as antifungal agents in vitro and compared to Ridomil, a reference chemical fungicide. The in vitro antifungal activity results revealed that Ridomil (2000 µg/mL) showed the highest fungal growth inhibition (FGI) against *A. alternata* (96.29%). Moderate activity was found against *A. alternata* by *D. viscosa* and *U. dioica* MLEs (2000 µg/mL), with an FGI value of 56.67 and 54.81%, respectively. The abundance of flavonoid and phenolic components were identified by HPLC analysis in the two plant extracts. The flavonoid compounds, including hesperidin, quercetin, and rutin were identified using HPLC in *D. viscosa* MLE with concentrations of 11.56, 10.04, and 5.14 µg/mL of extract and in *U. dioica* MLE with concentrations of 12.45, 9.21, and 5.23 µg/mL, respectively. α-Tocopherol and syringic acid, were also identified in *D. viscosa* MLE with concentrations of 26.13 and 13.69 µg/mL, and in *U. dioica* MLE, with values of 21.12 and 18.33 µg/mL, respectively. Finally, the bioactivity of plant extracts suggests that they play a crucial role as antifungal agents against *A. alternata*. Some phenolic chemicals, including coumaric acid, caffeic acid, ferulic acid, and *α*-tocopherol, have shown that they may be utilized as environmentally friendly fungicidal compounds.

## Introduction

Tomato (*Solanum lycopersicum* L.) is one of the world’s key important vegetable crops^1,2^. Fresh tomatoes suit the human body's fundamental nutritional needs as a functional food since they include a consistent amount of minerals and antioxidant chemicals like polyphenols^3^. Globally, tomato is the world’s largest produced vegetable crop with an annual creation of 182.26 million metric tons^4^. Tomato is sensitive to various diseases caused by bacteria, viruses, nematodes, fungi, and other pathogens^5^. Diseases induced by phytopathogenic fungi cause significant crop losses in tomato farming, accounting for up to 100% of the crop^6^.

*Alternaria* species continue to be an increasing hazard to a wide variety of crops around the world, producing a variety of illnesses. *Alternaria* disease produces significant yield losses and decreases the economic value of agricultural plants in present production methods^7^. Alternaria infections cause necrotic areas in circular rings with a yellow chlorotic halo, influencing plants by reducing the photosynthetic surface^8^. *A. solani* produces early blight disease in various solanaceous agricultural plant hosts^9,10^. Indeed, the presence of *A. alternata* tomato is often linked to the creation of mycotoxins including alternariol, altertoxin-I, II, alternariol methyl ether, and tenuazonic acid, which are all harmful to animal and human health^11^.

In Egypt, the symptoms of *A. alternata* leaf spot and blight have been commonly observed on tomatoes and potatoes^12^. *Alternaria* black spots are brown or black patches on leaves, stems, or pods that expand in warm, humid environments, reduce the photosynthetic area, defoliation, and accelerate the senescence^13^. Infection caused severe defoliation and significant production losses before flowering under ideal conditions^14^.

PCR was previously used to detect *Alternaria* spp. in tomatoes based on ribosomal internal transcribed spacer (ITS) DNA sequence analysis^15^. Meanwhile, ITS region sequencing revealed that *A. solani* and *A. alternata* species were the two most harmful isolated pathogens from tomato^16^. The two approaches addressed the inside transcribed spacer sections ITS1 and ITS2 of the rRNA gene, as well as a positive magnification control based on the 18S rRNA gene, using *Alternaria*-specific primers and probes^17^. Several investigators used other genes like *endoPG, Alt a1,* and *gapdh* for more accurate identification of *Alternaria* species^18,19^.

Chemical management of *Alternaria* infections improves crop output, but is ineffective and non-discriminatory in human and animal health and damages the environment^20,21^. As a result, numerous research has recommended employing ecologically friendly substances to counteract the spread of *Alternaria* diseases, such as biocontrol agents or extracts. *Urtica dioica L.* (popular nettle or sting nettle) plants are deemed medicinal herbs because of their pharmacological and natural properties^22,23^. The antifungal activity against *A. alternata* by all parts of the plant can be medicinally used; the leaves offer the most potent antioxidant, antibacterial, and anti-inflammatory properties^24,25^.

Caffeic and chlorogenic acids, β-sitosterol, and stigmasterol were detected in the extracts of *U. urens* and *U. dioica*^26^. Some other chemical compounds, such as volatile chemicals, lectins, terpenes, sterols, fatty acids, proteins, polysaccharides, vitamins, phenolics, and flavonoids were also detected in *U. dioica* extracts^23,27^. The ethyl acetate fraction extract of *U. dioica* had the most potent antimicrobial activity against *Aeromonas hydrophila*, *Salmonella typhi*, *Staphylococcus aureus*, *Bacillus cereus*, and *Escherichia coli*, with its highest content of polyphenols (48.3 mg GAE/gdw)^28^. *Staphylococcus* *aureus*, *B. subtilis*, and *Salmonella* spp. exhibited the greatest vulnerability to the antibacterial activity of *U. dioica* extracts^29^. Many herbal plants such as *U. dioica* are natural causes of mixtures with antibacterial, antifungal, and antioxidant effects^27^.

*Dodonaea viscosa* extracts showed antifungal activity against the three diseases that target commercial crops (*A. solani*, *Rhizoctonia solani*, and *Macrophomina phaseolina*)^30^. According to phytochemical screening, alkaloids, flavonoids, fixed oil and fat, steroids, phenolics, saponins, tannins, gums, mucillages, carbohydrates, reducing sugar, and glycosides were detected in *D. viscosa* extracts^31,32^. *D.* *viscosa* has antidiabetic, antimicrobial, insecticidal, antioxidant, cytotoxic, antifertility, wound, anti-inflammatory, analgesic, anti-ulcer, antispasmodic, anti-diarrheal, and detoxifying effects, according to pharmacological studies^31^. The HPLC investigation revealed the polyphenolic components i.e., rutin, gallic acid, catechin, caffeic acid, myricetin, and apigenin in various solvent extracts from *D. viscosa*^33^.

The study aimed to assess the ability of *D. viscosa,* and *U. Dioica* methanolic leaf extracts as an eco-friendly antifungal to suppress the *A. alternata* fungus.

## Materials and methods

### Isolation and purification of the fungal pathogen

This study has complied with relevant institutional, national, and international guidelines and legislation. This study does not contain any studies with human participants or animals performed by any of the authors, where one fungal isolate was retrieved from leaf spot symptoms on tomato plants at Rashid, El Behira governorate, Egypt. The isolation process was started with slicing the symptomatic leaves into small pieces (approx. 5 × 5 mm) and then surface-sterilized for 2 min with sodium hypochlorite solution (2%), ensued by excessive rinsing (2–3 times) with sterile dH_2_O. The small parts were put onto potato dextrose agar (PDA) media plates and then incubated at 25 ± 2 °C for a week. After that, the single spore method was used to obtain pure fungal culture, which was maintained in a fridge at 4 °C on PDA slants for additional studies.

### Pathogen’s recognition

#### Cultural and morphological features

A pathogen slide of the fungal isolate from pure cultures was produced and examined under a light microscope for identification. Isolated fungus morphological and cultural features were documented and compared to the standard keys to determine its identification^34^.

#### Molecular characterization of *Alternaria* isolate

Total genomic DNA was isolated from the mycelia of *Alternaria* isolate using CTAB (hexacetyl trimethyl ammonium bromide; Sigma-Aldrich, Germany) method^35^. The molecular identification of *A. alternata* using specific primers based on the internal transcripted spacer region (ITS) and small subunit ribosomal RNA gene (18SrRNA) gene as well as *endoPG*, *Alt a1* and *gapdh* genes.

The molecular identification was based on the internal transcripted spacer region (ITS) and small subunit ribosomal RNA gene (18SrRNA) gene. The ITS region was amplified with primers ITS1 (5′-TCCGTAGGTGAACCTGCGG-3′), ITS4 (5′-TCCTCCGCTTATTGATATGC-3′), and the 18SrRNA gene was amplified by the two primers NS1(5′-GTAGTCATATGCTTGTCTC-3′) and NS8 (5′-TCCGCAGGTTCACCTACGGA-3′)^36^. While the *endoPG* gene region was amplified using the primers PG3 (5′-TACCATGGTTCTTTCCGA-3′) and PG2b (5′-GAGAATTCRCARTCRTCYTGRTT-3′)^37^, Aalt For (5′-GTGCCTTCCCCCAAGGTCTCCG-3′) and Aalt Rev (5′-CGGAAACGAGGTGGTTCAGGTC-3′) was used for amplification of *Alt a1* gene region^38^, the last specific gene (*gapdh*) was amplified using two primers gab1(5′-CAACGGCTTCGGTCGCATTG-3′) and gpd2 (5′-GCCAAGCAGTTGGTTGTGC-3′) as described by Berbee et al.^39^. PCR reactions were done using 0.5 µL of each primer pair, 12.5 µL of 2 × *Taq* Ready Mix (Enzynomics Inc., Daejeon, Korea), and 1 µL of template DNA; then, the molecular grade water was up to a capacity of 25 µL. In a thermal cycler (Techne Prime, Cole-Parmer, Staffordshire, UK), a PCR program was performed as follows: pre-denaturation of 95 °C for 4 min, 35 cycles of three stages, 94 °C/45 s, annealing according to gene primer as described in references provided above, and 72 °C/1 min, and a final elongation step at 72 °C/5 min.

### Sequencing and phylogenetic analysis

The obtained ITS (~ 600 bp), 18SrRNA (~ 1500 bp), *endoPG* (~ 500 bp), *Alt a1* (~ 500 bp), and *gapdh* (~ 600 bp) amplicons of the selected isolate were delivered for sequencing (Macrogen Co, Seoul, Korea). The obtained sequences were equated to those in GenBank using the BLASTn tool. The alignment of ITS and 18SrRNA of *Alternaria* sequences were used to establish the phylogenetic trees compared with GenBank accessioned *Alternaria* isolates. The alignment of *endoPG*, *Alt a1,* and *gapdh* genes of *Alternaria* sequences was used to confirm the identification of *Alternaria* isolate and deposited in NCBI GenBank. The evolutionary history was implied using the highest parsimony method^40^.

### In vitro-evaluation of plant extracts versus the leaf spot pathogen equated to the chemical fungicide

Plant leaves from *U. dioica* and *D. viscosa* were gathered in Alexandria, Egypt. Under laboratory conditions, the samples were air-dried and pulverized in a tiny laboratory mill. Approximately 50 g of *U. dioica* and *D. viscosa* leaf samples were extracted for 3 days at room temperature with 200 mL of MeOH, then filtered using Whatman No. 1 filter paper^41^. The solvent was then evaporated, and the extracts were concentrated using a rotary evaporator at 45 °C under a vacuum. Furthermore, the crude extracts were kept in sealed vials at 4 °C until they were used for in vitro antimicrobial activity screening^41^. By dissolving the extract in dimethyl sulfoxide (DMSO 99.99%), the extracts and the chemical fungicide Ridomil (Metalaxyl-M (Mefenoxam)-Mancozeb, Germany) were prepared at concentrations of 100, 200, 300, 400, 500, 600, 700, 800, 900, 1000, 2000 µg/mL and tested against the growth of the isolated fungus using the food poison technique^42^. A 5 mm plug of the pure isolate was obtained from the borders of the pathogen's actively developing culture and put into each Petri plate. The Petri plates were then incubated at 25 ± 2 °C for 2 h. When the hyphal development in the control treatment was complete, mycelial growth was measured. Each treatment was given three times in total. The radial mycelial growth of the fungus was measured against both extracts, and the results were translated into the FGI % using a formula created by Naz et al.^43^ and were subjected to statistical analysis.

### HPLC analysis of phenolic and flavonoid components

The phenolic and flavonoid components from the methanol extracts of *U. dioica* and *D. viscosa* leaves were categorized by HPLC (Agilent 1100, USA). A binary LC pump, a UV/Vis detector, and a C18 column (125 mm, 4.60 mm, 5 m) make up this apparatus. The Agilent ChemStation was used to acquire and analyze chromatograms. A gradient mobile phase of two solvents—Solvent A (MeOH) and Solvent B [Acetic acid in H_2_O (1:25)]—was used to separate phenolic acids. The gradient program began with 100% B and remained there for 3 min. Thin was followed by 5 min of 50% eluent A, followed by 2 min of 80% eluent A, followed by 5 min of 50% eluent A, followed by 2 min of 80% eluent A, followed by 5 min of 50% eluent A, followed by 5 min of 50% eluent A, followed by 5 min of detection wavelength at 250 nm. As a result, the phenolic components were arranged in order to authenticate standard components by this mobile phase^44^.

### Antifungal assay of phenolic acids and mixtures

Based on the HPLC analysis of the studied extracts, the highest and more available phenolic acid compounds identified in both extracts were used for the bioassay against the growth of the isolated fungus by poisoned technique in the lab, as described previously in Sect. 2.4. The individual compounds and their combination/mixture with their exact percentage from the HPLC analysis were used against the growth of identified fungus.

### Statistical analysis

The experiment was statically analyzed by CoStat program ver., 6.303 (CoHort software, Monterey, CA, USA). A completely randomized design^45^ was performed and the means were equated by Duncan’s multiple range test^46^. The data were expressed as means ± SD values and were deemed statistically significant when *p* ≤ 0.05.

## Results

### Isolation and identification of the fungal pathogen

The symptoms of tomato leaf spot on field-infected plants yielded the same fungus strain from 20 leaves that showed the same signs. To identify the fungal isolate, morphological and molecular characterization were carried out.

#### Cultural and morphological traits

**Figure 1:**
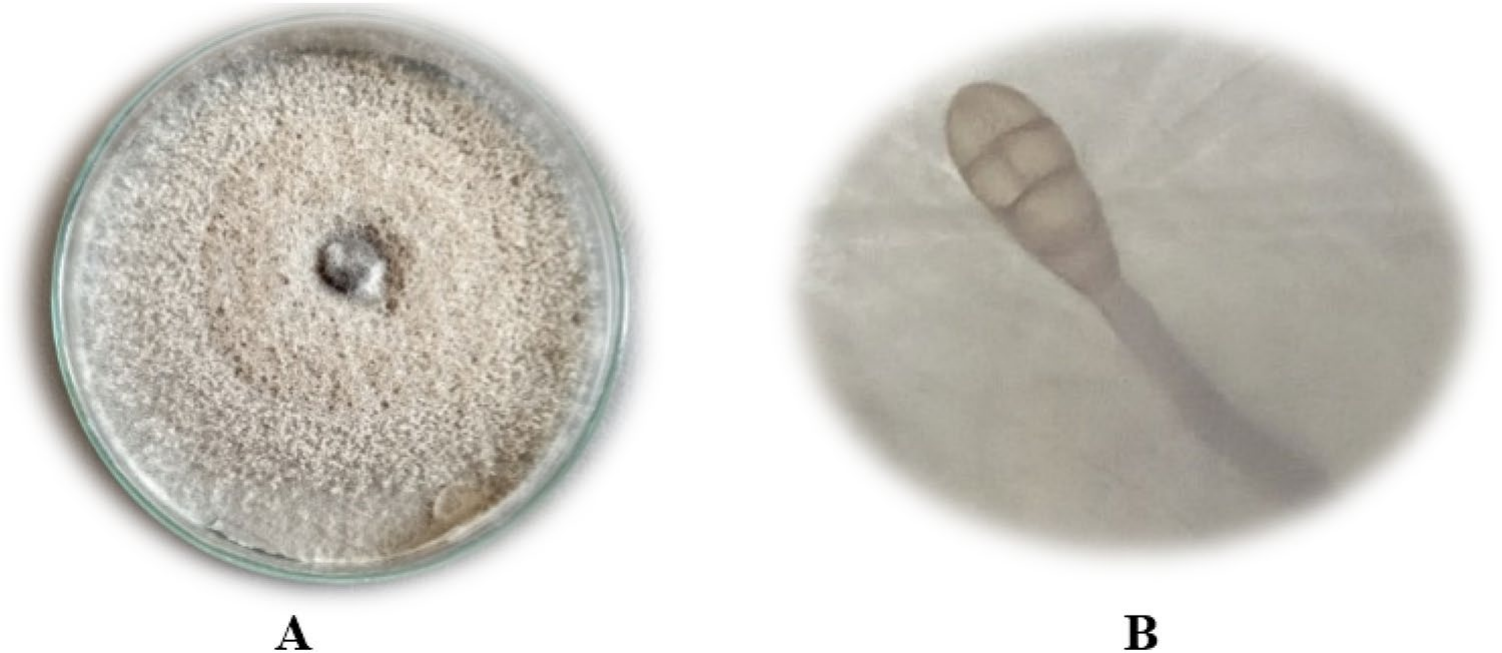
*Alternaria alternata* growth on a Petri dish (**A**) and conidiophore bearing the septated conidium was photographed under a light microscope 40x (**B**).

The PDA media plate fungal growth was smooth and deep brown or olivaceous brown (Fig. 1). According to microscopic features of the pathogenic fungus, the conidia were brown to olivaceous brown, solitary, straight, or ellipsoidal tapering, and possessed transverse and longitudinal septate. All these phenotypic traits aided in the initial identification of *Alternaria* sp.

#### Molecular genes analyses

The amplified sequences of ITS, 18SrRNA, *endoPG*, *Alt a1,* and *gapdh* genes of TAA-05 isolate were deposited in GenBank under accession numbers OL673807, OL674053, OP311598, OP311599, and OP311600, respectively. According to sequences data retrieved from aligning *endoPG, Alt a1,* and *gapdh* genes*,* the blasted results confirmed the identity of the fungal isolate as *A. alternata* isolate TAA_05 (Supplementary material).

**Figure 2:**
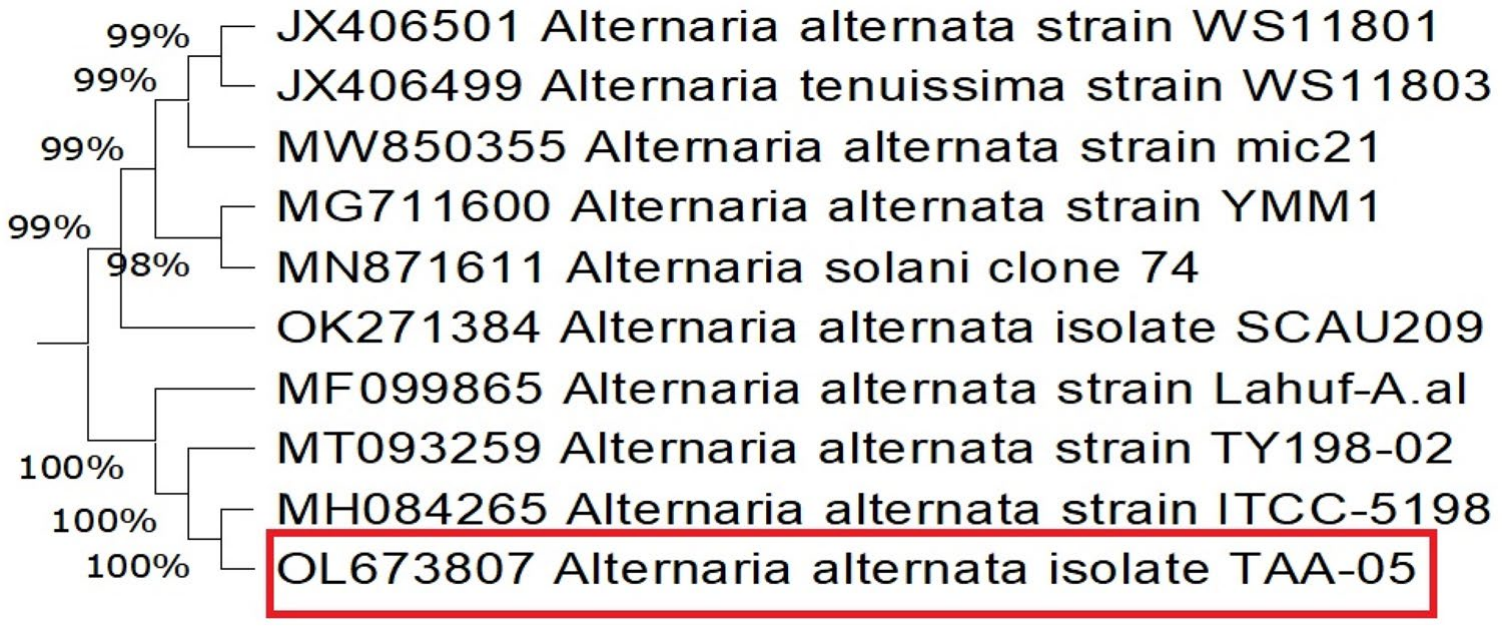
Constructed phylogenetic tree based on 10 most parsimonious ITS sequences. The Egyptian fungal isolate is *Alternaria alternata* TAA-05 (OL673807).

Our isolate TAA-05 showed the maximum homogeneity of 100% with *A. alternata* fungus, aligning different gene sequences with matched sequences in GenBank. The ITS phylogenetic tree showed a maximum nucleotide sequence similarity (100%) with *A. alternata* isolates from India (MH084265), China (MT093259), and Iraq (MF099865), a minimum nucleotide sequence similarity (99%) with *A. alternata* from China (JX406501), Egypt (MW850355) and *A. tenuissima* from China (JX406499) as shown in Fig. 2.

**Figure 3:**
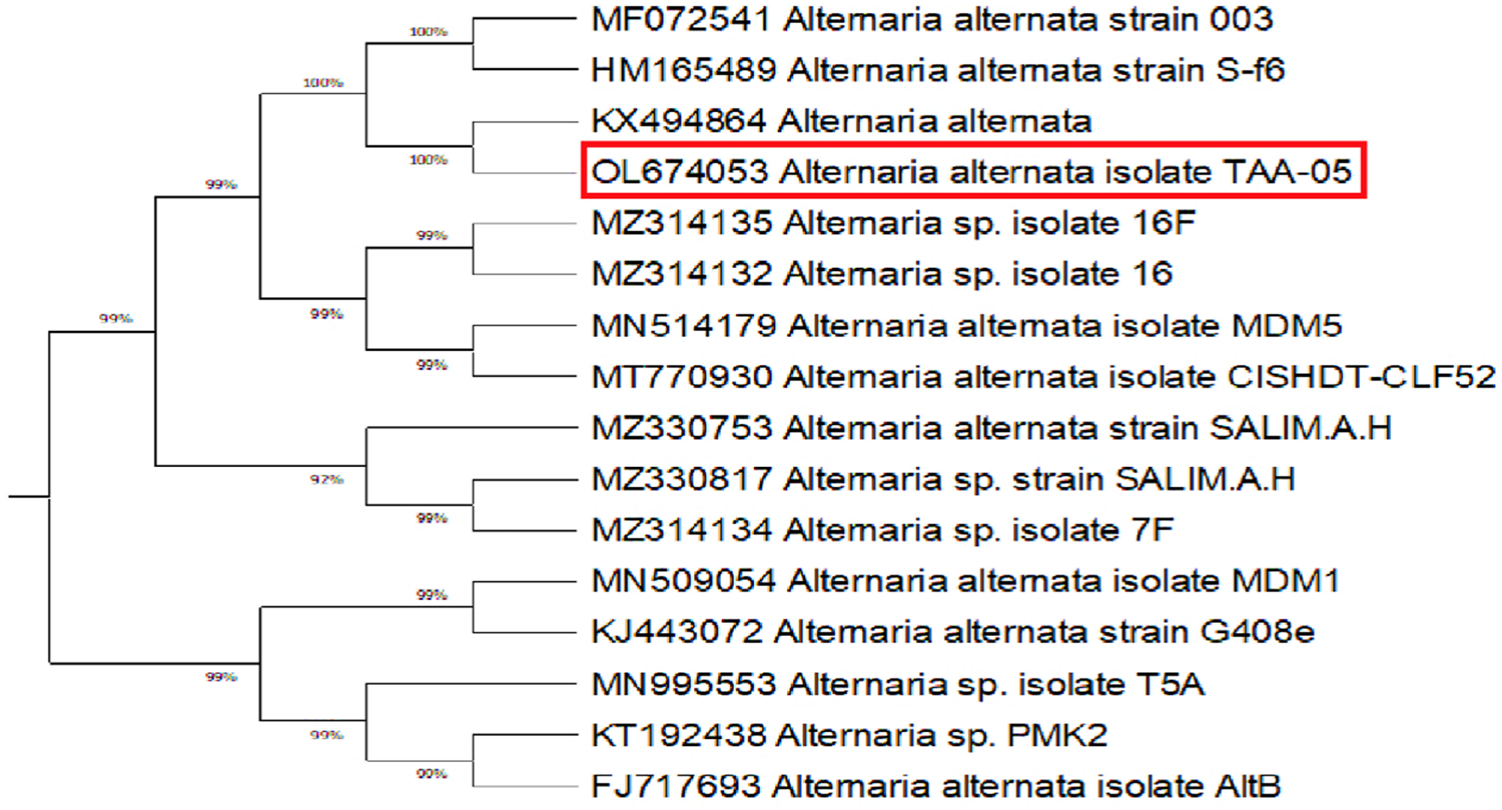
Constructed phylogenetic tree based on 16 most parsimonious 18SrRNA sequences. The Egyptian fungal isolate is *Alternaria alternata* TAA-05 (OL674053).

The 18SrRNA gene phylogenetic tree showed a maximum nucleotide sequence similarity (100%) with *A. alternata* isolates from India (KX494864), China (HM165489), and Portugal (MF072541). The minimum nucleotide sequence similarity (99%) with *A. alternata* from Saudi Arabia (MZ314132) and (MZ314135) is portrayed in Fig. 3.

### In vitro-evaluation of plant extracts versus *A. alternata*

**Figure 4:**
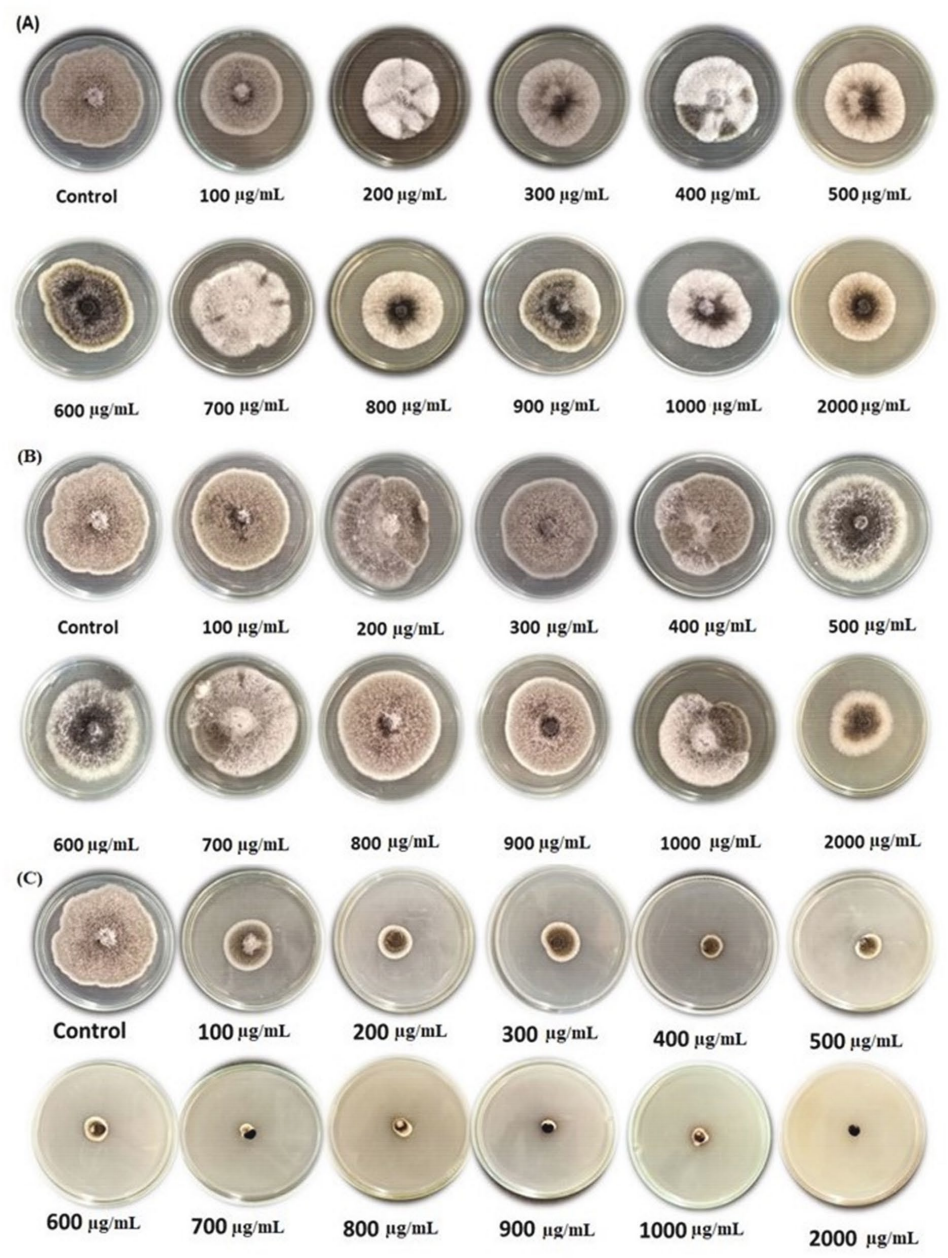
Visual observation of the antifungal activity of *U. dioica* extract (**A**), *D. viscosa* extract (**B**), and Ridomil fungicide (**C**) against the growth of *A. alternata* isolate.

The data tabulated in Table 1 show the extremely substantial impacts of *U. dioica* and *D. viscosa* methanolic leaf extracts (MLEs) against the growth of *A. alternata* isolate compared to the fungicide Ridomil as positive chemical control. At the level of concentrations from 100 to 2000 µg/mL, the inhibition percentages of *A. alternata* growth as affected by *U. dioica* MLE*, D. viscosa* MLE, and Ridomil fungicide were ranged between 38.89–54.81, 34.44–56.67, and 66.67–96.29%, respectively (Fig. 4).

**Table 1:** Antifungal activity of *U. dioica*, *D. viscosa* methanol extracts, and Ridomil fungicide on the growth of *A. alternata* isolate in vitro conditions.

Concentration (µg/mL)	Fungal growth inhibition (%) ± SD*		
	*U. dioica* MLE	*D. viscosa* MLE	Ridomil fungicide
0	0.00^w^	0.00^w^	0.00^w^
100	38.89^rs^ ± 0.08	34.44^v^ ± 0.08	66.67^h^ ± 0.08
200	39.63^qr^ ± 0.04	35.93^uv^ ± 0.12	77.41^g^ ± 0.04
300	40.00^qr^ ± 0.08	36.29^u^ ± 0.04	77.78^g^ ± 0.08
400	42.59^op^ ± 0.12	37.04^tu^ ± 0.04	84.44^f^ ± 0.08
500	43.70^no^ ± 0.09	37.41^stu^ ± 0.09	86.29^e^ ± 0.12
600	44.44^n^ ± 0.08	37.41^stu^ ± 0.04	88.52^d^ ± 0.04
700	46.29^m^ ± 0.04	37.41^stu^ ± 0.04	93.33^c^ ± 0.08
800	47.77^m^ ± 0.08	38.52^rst^ ± 0.04	93.70^bc^ ± 0.04
900	49.63^l^ ± 0.04	41.11^pq^ ± 0.08	94.44^bc^ ± 0.00
1000	51.85^k^ ± 0.12	46.67^m^ ± 0.16	95.18^ab^ ± 0.04
2000	54.81^j^ ± 0.12	56.67^i^ ± 0.08	96.29^a^ ± 0.04

*Standard deviation.

Means with the same letter/s are not significantly different according to LSD at 0.05 level of probability.

It is evident that the positive chemical control Ridomil fungicide was the most suppressive agent against *A. alternata* (TAA-05) isolate, with the highest mean growth reduction of 79.51%. Furthermore, the IFG value of the *U. dioica* MLE was 41.64%. With mean growth reduction values of 36.57%, the *D. viscosa* MLE was shown to have moderate activity (Table 1).

#### HPLC testing of flavonoids in *U. dioica* and *D. viscosa* extracts

**Figure 5:**
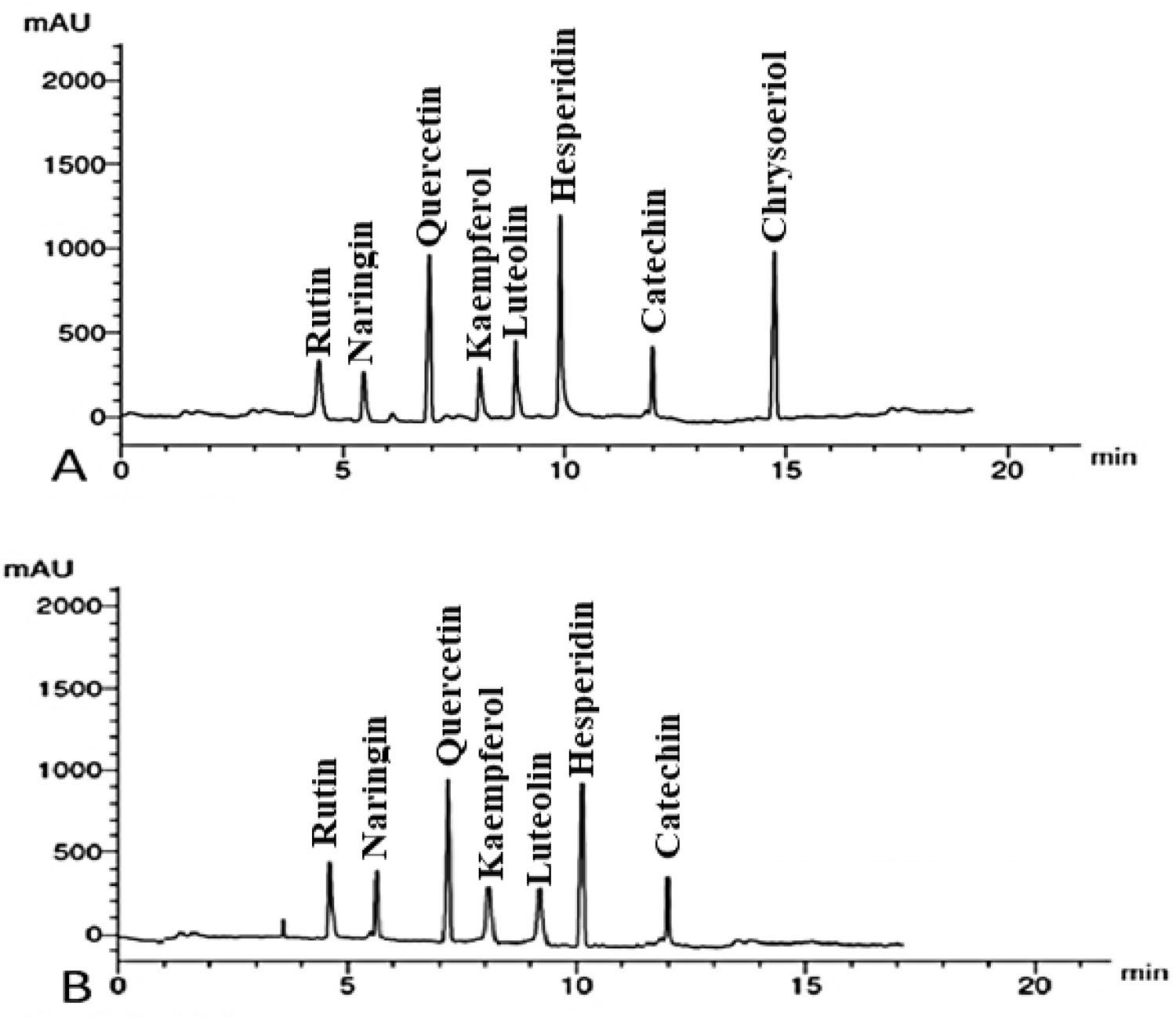
HPLC chromatograms of flavonoid compounds identified in the methanol leaf extracts of *U. dioica* (**A**) and *D. viscosa* (**B**).

The HPLC chromatograms of the flavonoids detected in *U. dioica* and *D. viscosa* MLEs are shown in Fig. 5. The abundant identified flavonoid compounds by µg/mL in *U. dioica* MLE were chrysoeriol (22.08), hesperidin (12.45), quercetin (9.21), and catechin (7.14). While in *D. viscosa* MLE were hesperidin (11.56), quercetin (10.04), and catechin (6.52) as indicated in Table 2.

**Table 2:** Flavonoid compounds identified in the methanolic leaf extracts from *U. dioica* and *D. viscosa* by HPLC.

Compound	Concentration (µg/mL) of flavonoid compounds in the methanol leaf extracts	
	*U. dioica*	*D. viscosa*
Rutin	5.23	5.14
Naringin	4.12	4.04
Quercetin	9.21	10.04
Kaempferol	4.05	3.17
Luteolin	6.09	2.88
Hesperidin	12.45	11.56
Catechin	7.14	6.52
Chrysoeriol	22.08	ND^*^

* Not detected.

#### HPLC analysis of phenolic components in *U. dioica* and *D. viscosa* leaf extracts

**Figure 6:**
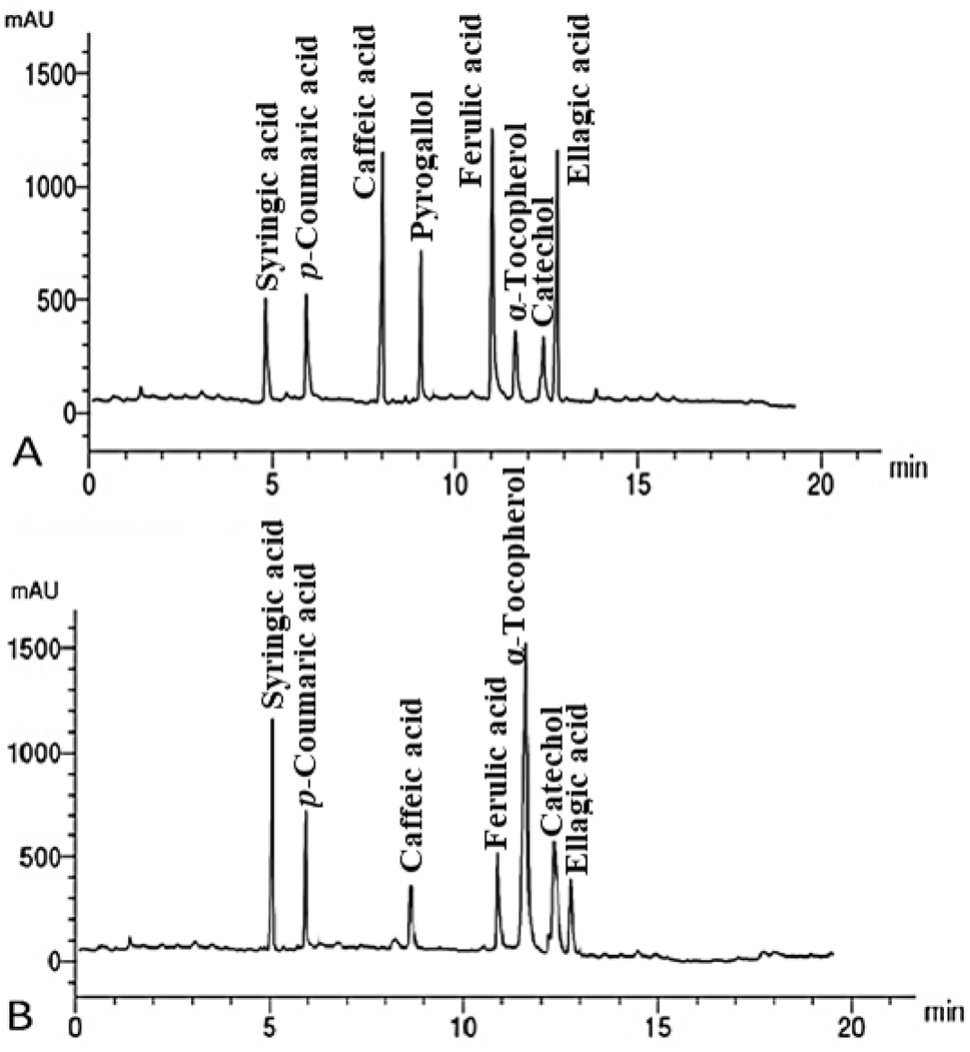
HPLC chromatograms of phenolic compounds identified in the methanolic leaf extracts of *U. dioica* (**A**) and *D. viscosa* (**B**).

The abundant phenolic compounds identified in *U. dioica* MLE by µg/mL were ferulic acid (21.12), caffeic acid (19.63), ellagic acid (18.33) and pyrogallol (14.51). While in *D. viscosa* MLE were α-tocopherol (26.13), syringic acid (13.69), and *p*-coumaric acid (10.11) as presented in Table 3 and Fig. 6.

**Table 3:** Phenolic compounds identified in the leaf methanol extracts from *U. dioica* and *D. viscosa* by HPLC.

Compound	Concentration (µg/mL) in the methanol leaf extracts	
	*U. dioica*	*D. viscosa*
Syringic acid	8.66	13.69
*p*-Coumaric acid	8.14	10.11
Caffeic acid	19.63	5.44
Pyrogallol	14.51	ND^*^
Ferulic acid	21.12	7.12
α-Tocopherol	7.45	26.13
Catechol	5.18	7.23
Ellagic acid	18.33	4.14

*Not detected.

#### Antifungal assay of phenolic acids and mixtures

According to the HPLC analysis of phenolic compounds, percentages results; the following phenolic compounds (coumaric acid, caffeic acid, ferulic acid, and α-tocopherol) were chosen to assess their antifungal activity against *A. alternata* alone or in mixes (the phenolic compounds concentrations of each extract shown in Table 3 were used). All the treatments are listed in Table 4.

**Table 4:** The most abundant phenolic compounds identified in the leaf methanol extract from *U. dioica* and *D. viscosa* by HPLC and their mixtures used in vitro against *A. alternata* isolate.

Compounds	Fungal growth inhibition (%)	
	*U. dioica* MLE	*D. viscosa* MLE
Control	0.00^m^	0.00^m^
Positive control (Ridomil)	83.33^a^ ± 1.11	83.33^a^ ± 1.11
Ferulic acid	55.18^i^ ± 1.69	55.92^g,h^ ± 0.64
Coumaric acid	40.00^k,l^ ± 1.11	50.00^i^ ± 1.11
Caffeic acid	50.00^i^ ± 1.11	43.33^j^ ± 1.11
α-Tocopherol	44.4^j^ ± 1.11	44.07^j^ ± 1.69
Ferulic + coumaric acids	51.11^i^ ± 1.11	67.03^b^ ± 0.64
Ferulic + caffeic acids	60.74^c,d,e^ ± 0.64	58.88^e,f^ ± 2.93
Ferulic acid + α-tocopherol	60.00^d,e,f^ ± 1.11	61.11^c,d,e^ ± 1.11
Caffeic + coumaric acids	58.14^f,g^ ± 1.69	61.48^c,d^ ± 1.69
Caffeic acid + α-tocopherol	39.25^l^ ± 0.64	42.59^j,k^ ± 1.69
Coumaric acid + α-tocopherol	40.37^k^ ± 1.69	55.18^h^ ± 1.69
Ferulic acid + coumaric acid + caffeic acid + α-tocopherol	69.25^b^ ± 1.69	62.96^c^ ± 1.69

Means with the same letter are not significantly different according to LSD at 0.05 level of probability.

**Figure 7:**
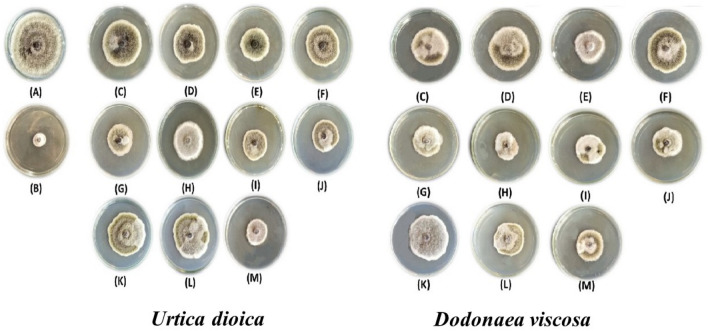
Antifungal activity of *Urtica dioica* and *Dodonaea viscosa* most abundant compounds and mixtures. A = Control, B = Positive control (Ridomil), C = Caffeic acid, D = Ferulic acid, E = α-Tocopherol, F = Ferulic acid + caffeic acid, G = Ferulic acid + coumaric acid, H = Ferulic acid + α-tocopherol, I = Caffeic acid + coumaric acid, J = Caffeic acid + α-tocopherol, K = Coumaric acid + α-tocopherol, L = Coumaric acid, M = Total mixture.

According to the previous results from HPLC analysis, Table 4 shows the significant effects of *U. dioica* and *D. viscosa* MLEs against the growth of *A. alternata* isolate compared to the fungicide Ridomil as a positive chemical control in vitro. It is evident that the positive chemical control Ridomil fungicide was the most suppressive agent against *A. alternata* (TAA-05) isolate, with the highest mean growth reduction of 83.33%. In addition, the total combinations of *U. dioica* MLE were found to have a high FGI value of 69.25%, followed by ferulic and caffeic acids combination of 60.74%. The highest significance was shown with ferulic and coumaric acids of *D. viscosa* MLE was applied with mean growth reduction values of 67.03%, followed by total combinations of 62.96%. On the other hand, the lowest significance showed with caffeic acid and α-tocopherol combinations of *U. dioica* MLE, and *D. viscosa* MLE was found to have low FGI values of 39.25 and 42.59%, respectively (Fig. 7).

## Discussion

Leaf spot disease caused by *A. alternata* is a significant disease in tomato and potato^47,48^. Our study investigated one fungal isolate of the leaf spot disease pathogen of *A. alternata* (TAA-05). The fungal strain was identified morphologically using Simmons' morphological parameters, including outpost morphology, size, conidia shape, and conidial septation pattern^49^.

The attributes of this pathogen were reliable with the features depicted by the Commonwealth Mycological Institute, Kew, Surrey, England^50^. Therefore, the pathogen was recognized as *A. Alternata*^51^, based on the morphological characteristics, ITS region, 18SrRNA, *endoPG*, *Alt* a1, and *gapdh* genes amplification and sequencing. Based on earlier findings, *A. alternata* is the crucial reason for black spot blight symptoms in the family Solanaceae^52^. Additionally, molecular methods are appropriate assays for analysis, especially for scientists unfamiliar with the traditional features of fungi^53^. Sequencing the ITS region of ribosomal DNA, which identifies *Alternaria* spp. from other infections quite well^49,50^, is one of these approaches. The morphological characterization of the examined fungal isolates acquired in this study was corroborated by data collected from genetic studies^54,55^.

The antifungal effect of dual plant extracts *U. dioica* and *D. viscosa* were assayed against *A. alternata* growth. Both plants extract with a range of 100 to 2000 µg/mL were exhibited an antifungal possibility versus the pathogenic fungus. Previously, *U. dioica* extract inhibited *A. alternata* growth by 59.78 and 77.81%, at 1000 and 1500 µg/mL, respectively^24^. These findings revealed that *U. dioica* extract was highly effective against *A. alternata* and *R. solani*, suggesting that it might be used as a chemical additive to manage fungal infections in plants^56^.

*D.* *viscosa* was revealed to be highly efficient, suppressing the radial mycelial growth of *A. solani* (56.96%), *Macrophomina phaseolina* (52.06%), and *Rhizoctonia solani* (51.54%)^57^. *D.* *viscosa* extract significantly suppressed the circular mycelial growing of *A. solani* with a value of 56.96%^30^. While other study showed inhibition values of 68.48%, in in vitro and in vivo conditions^58^.

*D.* *viscosa* extract at 300 μL showed the maximum scavenging activity with an inhibition rate of 82.09%, observed by 200 μL with values of 81.02%, and 100 μL with a rate of 79.91%^59^. The most excellent extracts with > 50% reticence against *A. solani* were *Elaeagnus angustifolia*, *D. viscosa*, *Haplophyllum perforatum*, and inflorescence of *Allium hirtifolium*, respectively^60^.

The abundant identified flavonoid compounds from *U. dioica* MLE were chrysoeriol, hesperidin, quercetin and catechin. While in *D. viscosa* MLE were hesperidin, quercetin, and catechin. The abundant phenolic components identified in the *U. dioica* MLE were ferulic acid, caffeic acid, ellagic acid, and pyrogallol, while in *D. viscosa* MLE were ferulic, ellagic, syringic, and *p*-coumaric acids. The flavonoid compounds identified in the *U. dioica* MLE were rutin, naringin, quercetin, kaempferol, luteolin, hesperidin, catechin, and chrysoeriol, while all of them were identified except chrysoeriol in *D. viscosa* MLE. Phenolic compounds of acids of syringic, *p*-coumaric, caffeic, ferulic, and ellagic, as well as pyrogallol, α-tocopherol, and catechol were identified in both extracts. Flavonoids and diterpenoids are the strongest secondary metabolites formerly recognized and separated from *D. viscosa*^61^.

Several chemical groups were identified in *D. viscosa* extracts like alkaloids, flavonoids, phenolics, steroids, saponins, tannins, and gums^31^. In the several plant sections of *D. viscosa*, HPLC–DAD examination found considerable amounts of rutin, vanillic acid, coumaric acid, ferulic acid, gallic acid, syringic acid, cinnamic acid, gentisic acid, catechin, caffeic acid, apigenin, and myricetin^33^*.* Furthermore, cafeoil-malic acid, chlorogenic acid, ferulic acid, rutin, isoquercitrin ,and astragalin were identified in *U. dioica* and their antioxidant activity was reported^62^.

*U. dioica* LE collected in March had the greatest levels of polyphenolcarboxilic acids (expressed as a percentage of chlorogenic acid) and flavonoids (expressed as a percentage of rutin) (4.2295% chlorogenic acid and 0.6237% rutin)^63^. The *p*-hydroxybenzoic acid, gentisic acid, quinic acid, protocatechuic vanillic acid, caffeic acid, ferulic acid, 5-O-caffeoylquinic, esculetin, scopoletin, chrysoeriol, α-sitosterol, scopoletin are polyphenols found in extracts from *U. dioica*^64^. Polyphenols also are included isorhamnetin, kaempferol, kaempferol plastocyanins, quercitrin, glycoproteins, rutin, amentofavon, 3-O-glucoside, quercetin 3-O-glucoside, plastocyanins, quercitrin, glycoproteins, rutin, and amentofavon^65^. Caffeic acid, chlorogenic acid, -sitosterol, stigmasterol, rutin, and ergosterol were found in *U. dioica*, according to the chromatographic data^26^. The methanolic extracts of *U. dioica* showed a synergistic effect in mixture with an inhibition effect on bacteria and fungi antibiotics. Furthermore, HPTLC showed amounts of phenolics, tannin, flavonoids, carbohydrates, glycosides, and saponins were detected in *U. dioica* MeOH-extracts^66^.

Extracts containing flavonoids and phenolic compounds have been intended to suppress fungal diseases by inhibiting the germination of fungal spores^44,67–72^. Flavonoids inhibit many types of eukaryotic enzymes. This suppression may be due may be owing to the enzyme interactions with various portions of the flavonoid molecule, i.e., carbohydrates, phenyl rings, phenols, and benzopyrone rings^73^.

Using the higher abundant compounds of *U. dioica* and *D. viscosa,* MLEs showed the most elevated significance from the mixture combinations of *U. dioica* and the mixture of ferulic and coumaric acids of *U. dioica*. In contrast, mixture compounds from *D. viscosa* MLE followed by ferulic and coumaric compounds showed the highest significance.

Phenolic acids (ferulic and *p*-coumaric) were also estimated. In a synthetic medium, ferulic and *p*-coumaric acids had considerably increased inhibitory capability; ferulic acid was continued active against *M. fructicola* and *A. alternata* and was more effective than *p*-coumaric acid in controlling *B. cinerea*^47^. Phenolic compounds, like caffeic, 2,3,4-trihydroxybenzoic, p-coumaric, and protocatechuic acids achieved the highest antifungal effects and almost wholly inhibited *A. alternata* on early and late-ripening sweet cherries^74^. Furthermore, 4 phenolic components *i.e.,* salicylic acid, catechol, trans-cinnamic acid, and *p*-coumaric acid, prevented the expansion of *A. solani* more efficiently than others^75^. *p*-Coumaric acid also totally inhibited the *Fusarium* growth. In contrast, ferulic acid prevented 64% of *Fusarium* mycelial growing at the same dose of 1000 μg/g and was particularly efficient against *B. cinerea* growth^76,77^. Vanillic and caffeic acids (0.2 mg/mL) suppressed the development and formation of aflatoxin by *A. flavus* and *parasiticus*. In comparison, 0.3 mg/mL of *p*-hydroxy benzoic, protocatechuic, syringic, and *p*-coumaric acids, and quercetin completely inhibited the molds (Supplementary information)^78^.

## Conclusion

Herein, the methanolic leaf extracts of *U. dioica* and *D. viscosa* demonstrated a strong antifungal efficacy against the *A. alternata* pathogen, the causal agent of tomato leaf spot disease. Among many polyphenolic compounds that were detected in the HPLC of the two extracts, coumaric acid, caffeic acid, ferulic acid, and α-tocopherol showed potent in vitro fungicidal activity against *A. alternata,* either applied alone or in combination at low concentrations. Consequently, the application of polyphenolic compounds could offer an alternative way, environmentally safe and economically acceptable, for the management of plant fungal diseases. However, additional research is required to corroborate these results in the field.

## Supplementary Information

Supplementary Information.

## Data Availability

All data generated or analyzed during this study are included in this published article.
