# Supplementary material for: Urtica dioica and Dodonaea viscosa leaf extracts as eco-friendly bioagents against Alternaria alternata isolate TAA-05 from tomato plant
Source: Sci Rep. 2022 Oct 1;12:16468. doi: 10.1038/s41598-022-20708-4 (PMC9526714; doi:10.1038/s41598-022-20708-4)
Supplement: Supplementary file 1 — Supplementary Information. [file 41598_2022_20708_MOESM1_ESM.docx]

***Urtica dioica* and *Dodonaea viscosa* leaf extracts as eco-friendly bioagents against *Alternaria alternata* isolate TAA-05 from tomato plant**

**Said I. Behiry^1^, Bassant Philip^1^, Mohamed Z. M. Salem^2,^*, Mostafa A. Amer^1^, Ibrahim A. El-Samra^1^, Ahmed Abdelkhalek^3^, and Ahmed Heflish^1^**

^1^Agricultural Botany Department, Faculty of Agriculture (Saba Basha), Alexandria University, Alexandria 21531, Egypt

^2^ Forestry and Wood Technology Department, Faculty of Agriculture (El-Shatby), Alexandria University, Alexandria 21545, Egypt

^3^Plant Protection and Biomolecular Diagnosis Department, ALCRI, City of Scientific Research and Technological Applications, New Borg El Arab City 21934, Egypt

*: Corresponding author: [mohamed-salem@alexu.edu.eg](mailto:mohamed-salem@alexu.edu.eg)

**Nucleotide Sequence**

LOCUS OP311598 491 bp DNA linear PLN 21-AUG-2022

DEFINITION Alternaria alternata.

ACCESSION OP311598

VERSION

KEYWORDS .

SOURCE Alternaria alternata

ORGANISM Alternaria alternata

Eukaryota; Fungi; Dikarya; Ascomycota; Pezizomycotina;

Dothideomycetes; Pleosporomycetidae; Pleosporales; Pleosporineae;

Pleosporaceae; Alternaria; Alternaria sect. Alternaria; Alternaria

alternata complex.

REFERENCE 1 (bases 1 to 491)

AUTHORS Behiry,S.I., Abdelkhalek,A., Heflish,A.A., Salim,M., Amer,M. and

Elsamra,I.

TITLE Direct Submission

JOURNAL Submitted (21-AUG-2022) Agricultural Botany, Faculty of Agriculture

Saba Basha, Tag elroasaa, Alexandria, 21531, Egypt

COMMENT Bankit Comment: TOTAL # OF SEQS:5

##Assembly-Data-START##

Sequencing Technology :: Sanger dideoxy sequencing

##Assembly-Data-END##

FEATURES Location/Qualifiers

source 1..491

/organism="Alternaria alternata"

/mol_type="genomic DNA"

/host="Tomato"

/db_xref="taxon:5599"

/country="Egypt"

/collected_by="Said Behiry"

gene <1..>491

/gene="endoPG"

CDS <1..>491

/gene="endoPG"

/codon_start=3

/product="endopolygalacturonase"

/translation="TMVLSDVAVPSGTTLDLSSLADGTTVIFEGTTTWGYSEWKGPLL

DIQGKKITVKGAEGSVLNGDGARWWDGKGGNGGKTKPKFFSAHKLTDSTITGITIKNP

PVQVVSINGCDGLTITDMTIDASDGDKDEQGHNTDGFDIGSSNNVIIDGAKVYNQDDC

EFS"

BASE COUNT 121 a 135 c 123 g 112 t

ORIGIN

1 ttaccatggt tctttccgac gttgccgtcc cttcaggcac aactttggac ctctctagtc

61 tggctgacgg tactactgtc atcttcgagg gtaccaccac ctggggctac tcggaatgga

121 agggtcccct tcttgacatc caaggaaaga agatcactgt caagggcgcc gagggatctg

181 ttctcaacgg tgatggtgct cgttggtggg acggtaaggg tggaaatggt ggaaagacca

241 agcccaagtt cttctccgct cacaaactga ccgactccac catcaccggc attaccatca

301 agaaccctcc cgtccaagtc gttagtatca acggctgcga tggtcttacc attacagaca

361 tgactattga tgcgtccgac ggcgacaagg acgagcaggg ccacaacaca gatggtttcg

421 atattggctc cagcaacaac gtcatcattg atggcgctaa ggtttacaac caagacgact

481 gcgaattctc a

//

LOCUS OP311599 478 bp DNA linear PLN 21-AUG-2022

DEFINITION Alternaria alternata.

ACCESSION OP311599

VERSION

KEYWORDS .

SOURCE Alternaria alternata

ORGANISM Alternaria alternata

Eukaryota; Fungi; Dikarya; Ascomycota; Pezizomycotina;

Dothideomycetes; Pleosporomycetidae; Pleosporales; Pleosporineae;

Pleosporaceae; Alternaria; Alternaria sect. Alternaria; Alternaria

alternata complex.

REFERENCE 1 (bases 1 to 478)

AUTHORS Behiry,S.I., Abdelkhalek,A., Heflish,A.A., Salim,M., Amer,M. and

Elsamra,I.

TITLE Direct Submission

JOURNAL Submitted (21-AUG-2022) Agricultural Botany, Faculty of Agriculture

Saba Basha, Tag elroasaa, Alexandria, 21531, Egypt

COMMENT Bankit Comment: TOTAL # OF SEQS:5

##Assembly-Data-START##

Sequencing Technology :: Sanger dideoxy sequencing

##Assembly-Data-END##

FEATURES Location/Qualifiers

source 1..478

/organism="Alternaria alternata"

/mol_type="genomic DNA"

/host="Tomato"

/db_xref="taxon:5599"

/country="Egypt"

/collected_by="Said Behiry"

gene <1..>478

/gene="Alt a 1"

CDS join(<1..323,384..>478)

/gene="Alt a 1"

/codon_start=1

/product="Alt a 1 major allergen"

/translation="ASLFAAAGLAAAAPLESRQDTASCPVTTEGDYVWKISEFYGRKP

EGTYYNSLGFNIKATNGGTLDFTCSHSADKLEDHTWYSCGENSFMDFSFDSDRNGLLL

KQKVSDSITYVATATLPNYCRAGGNGPKDFVCQGVAD"

BASE COUNT 101 a 159 c 112 g 106 t

ORIGIN

1 gcctctctct tcgccgccgc tggccttgcc gccgctgcac ctctcgagtc tcgccaggac

61 accgcatcct gccctgtcac caccgagggt gactacgtct ggaagatttc cgagttctac

121 ggacgcaagc cggagggaac ctactacaac agcctcggct tcaacatcaa ggctaccaac

181 ggaggaacac tcgacttcac ctgctctcac tcagccgaca agcttgagga ccacacttgg

241 tactcttgcg gcgagaacag cttcatggac ttctctttcg acagcgaccg caacggtctg

301 ctcctgaagc agaaggttag cgacgagtaa gttacccttg taccttcgat tacttcgcag

361 attcagatat actaacatgt ttccagcatc acctatgtcg ctaccgccac tcttcccaac

421 tactgccgcg ctggcggtaa cggccctaag gactttgtct gccagggtgt tgccgacg

//

LOCUS OP311600 555 bp DNA linear PLN 21-AUG-2022

DEFINITION Alternaria alternata.

ACCESSION OP311600

VERSION

KEYWORDS .

SOURCE Alternaria alternata

ORGANISM Alternaria alternata

Eukaryota; Fungi; Dikarya; Ascomycota; Pezizomycotina;

Dothideomycetes; Pleosporomycetidae; Pleosporales; Pleosporineae;

Pleosporaceae; Alternaria; Alternaria sect. Alternaria; Alternaria

alternata complex.

REFERENCE 1 (bases 1 to 555)

AUTHORS Behiry,S.I., Abdelkhalek,A., Heflish,A.A., Salim,M., Amer,M. and

Elsamra,I.

TITLE Direct Submission

JOURNAL Submitted (21-AUG-2022) Agricultural Botany, Faculty of Agriculture

Saba Basha, Tag elroasaa, Alexandria, 21531, Egypt

COMMENT Bankit Comment: TOTAL # OF SEQS:5

##Assembly-Data-START##

Sequencing Technology :: Sanger dideoxy sequencing

##Assembly-Data-END##

FEATURES Location/Qualifiers

source 1..555

/organism="Alternaria alternata"

/mol_type="genomic DNA"

/host="Tomato"

/db_xref="taxon:5599"

/country="Egypt"

/collected_by="Said Behiry"

gene <1..>553

/gene="gapdh"

CDS join(<1..18,74..136,250..>553)

/gene="gapdh"

/codon_start=3

/product="glyceraldehyde-3-phosphate dehydrogenase"

/translation="IVFRNGIEHNDVDIVAVNDPFIEPHYEAYMLKYDSTHGQFKGEI

KVDGNNLTVNGKTIRFHMEKDPANIPWSETGAYYVVESTGVFTTTEKAKAHLKGGAKK

VVISAPSADAPMFVMGVNHETYKSD"

BASE COUNT 139 a 178 c 130 g 108 t

ORIGIN

1 gtatcgtctt ccgcaatgcg taagtttcgc ctaactcgtc gatacaatct accagagctg

61 accgcatgcc acagtatcga gcacaacgac gtcgacattg tcgccgtaaa cgaccccttc

121 atcgagcccc actacgctgt aagcttcccc aagcacccac actacagccg cggccatcca

181 agttgcgaaa acagtccttg cgatgcgcta gagctcctct gtggtcgcag aatgcaggct

241 aacacattca ggcctacatg ctcaagtatg acagcacaca cggccagttc aagggtgaga

301 tcaaggttga cggcaacaac ctgaccgtca acggcaagac catccgtttc cacatggaga

361 aggaccccgc caacatccca tggagcgaga ccggcgctta ctacgtcgtc gagtccaccg

421 gtgtcttcac caccaccgag aaggccaagg ctcacttgaa gggtggagcc aagaaggtcg

481 tcatttctgc tccctctgct gacgccccca tgttcgttat gggtgtcaac cacgagactt

541 acaagtctga catcg

//
